# Pursuing Polymer Dielectric Interfacial Effect in Organic Transistors for Photosensing Performance Optimization

**DOI:** 10.1002/advs.201700442

**Published:** 2017-10-16

**Authors:** Xiaohan Wu, Yingli Chu, Rui Liu, Howard E. Katz, Jia Huang

**Affiliations:** ^1^ School of Material Science and Engineering Tongji University Shanghai 201804 P. R. China; ^2^ Department of Materials Science and Engineering Johns Hopkins University 3400 North Charles Street Baltimore MD 21218 USA

**Keywords:** interfacial effect, organic field‐effect transistor, photosensor, polymer dielectric, synaptic‐emulating devices

## Abstract

Polymer dielectrics in organic field‐effect transistors (OFETs) are essential to provide the devices with overall flexibility, stretchability, and printability and simultaneously introduce charge interaction on the interface with organic semiconductors (OSCs). The interfacial effect between various polymer dielectrics and OSCs significantly and intricately influences device performance. However, understanding of this effect is limited because the interface is buried and the interfacial charge interaction is difficult to stimulate and characterize. Here, this challenge is overcome by utilizing illumination to stimulate the interfacial effect in various OFETs and to characterize the responses of the effect by measuring photoinduced changes of the OFETs performances. This systemic investigation reveals the mechanism of the intricate interfacial effect in detail, and mathematically explains how the photosensitive OFETs characteristics are determined by parameters including polar group of the polymer dielectric and the OSC side chain. By utilizing this mechanism, performance of organic electronics can be precisely controlled and optimized. OFETs with strong interfacial effect can also show a signal additivity caused by repeated light pulses, which is applicable for photostimulated synapse emulator. Therefore, this work enlightens a detailed understanding on the interface effect and provides novel strategies for optimizing OFET photosensory performances.

## 1 Introduction

Flexible and stretchable organic field‐effect transistors (OFETs) have attracted intensive research and commercial interests since they are applicable in wearable devices, biomedical electronics, and various sensors.^1^, ^2^, ^3^, ^4^ Polymer dielectrics are essential for providing these devices with overall flexibility, stretchability, printability, and in some cases biocompatibility.^1^, ^4^, ^5^, ^6^, ^7^ Microstructural pattern designs on OFET dielectrics for achieving high performance devices have been realized by using polymer insulators too.^8^, ^9^, ^10^ Polymer dielectrics, with a variety of types, are thus extensively employed in OFETs. In such devices, charge carriers of organic semiconductors (OSCs) are routinely found partially trapped near the polymer dielectric interface.^7^, ^11^, ^12^, ^13^ OFETs with the same OSC but different polymer dielectrics could exhibit considerably different performances, indicating the interfacial charge interaction is intricate.^13^, ^14^ Moreover, the conduction of charge carriers in OFETs are usually concentrated within a couple molecular layers at the bottom of the OSC film near the dielectric interface. Thus, the interfacial effect possesses significant influence on the device characteristics.^15^, ^16^ However, understanding of the intricate and important interfacial effect is still limited, because the interface is buried under the OFETs surface and the charge interaction is difficult to stimulate and characterize.

Previous efforts have been made to study the “hidden” interfacial effect. For example, Scanning Kelvin probe microscopy was employed to directly observe the charge protein on the interfaces in lateral organic transistors.^17^, ^18^, ^19^ OFETs with OSC single crystals and a parylene dielectric were also used to study photoinduced charge transfer across the interface.^20^, ^21^ Several profiles of the interfacial effect were revealed based on these investigations, primarily including: (a) shallow trap of charge carriers in OSC by the weak intermolecular interaction from the dielectric, leading to a mobility (μ) decrease for the OFET,^22^ and (b) transfer of charge carriers across the interface from OSC to the dielectric and a deep trap by functional groups in the polymer, leading to a threshold voltage (*V*_th_) change and bias stress effect for the device.^17^, ^23^ Although these works provided basic mechanism for the interfacial effect, there are still much to be understood in the research field. First, more and more OSCs and polymer dielectric materials are applied in OFETs, but the general concept on how the molecular characteristics of the materials affect the interfacial effect is lacking; Second, the inducements and processes of the shallow and deep traps in OFETs remain unclear, as well as the dependency on the two trap models.

The interfacial effect was found to be more or less responsive to light.^11^, ^12^ In this work, we utilized illumination to stimulate and amplify the interfacial effect in OFETs with a range of OSCs and polymer dielectrics, and characterized the response of the effect by measuring photo‐induced changes of the OFETs characteristics. We found that the photosensitive OFET characteristics, including drain–source current (*I*_DS_), μ, and *V*_th_, were significantly affected by polar group of the polymer dielectrics and the OSC side chain. Polymer dielectrics with moderate polar groups provided the OFETs with dramatically photosensitive μ, while OSCs without side chains leads to devices with pronounced photosensitive μ and *V*_th_. Based on these results, a mechanism model was proposed that mathematically describes how the molecular characteristics of OSCs and polymer dielectrics influence shallow traps and deep traps on the interface. Moreover, OFETs with strong interfacial effect show an excitatory postsynaptic current (EPSC)‐like photoresponse behavior, such as long term of current recovery when the light exposing on the devices is turned off, and photocurrent additivity caused by repeated light pulses. These features make the OFETs applicable for photostimulated synapse emulator, which is promising for brain‐inspired electronics and human–machine interface, etc.,^8^ various photosensors with controllable performances were also obtained by utilizing this mechanism. For instance, the light‐to‐dark current ratio (*I*_light_/*I*_dark_) of the devices can be tuned in a wide range from below 10 to beyond 10^4^. Therefore, this work enlightens a comprehensive understanding on the interfacial effect, which enables prediction of OFETs characteristics with given component OSCs and dielectrics. We also provide novel strategies to simply fabricate synapse‐emulating OFETs and photosensors with controllable performances.

## 2 Results and Discussion

### 2.1 Stimulation of Illumination to the Interfacial Effect

**Figure 1:**
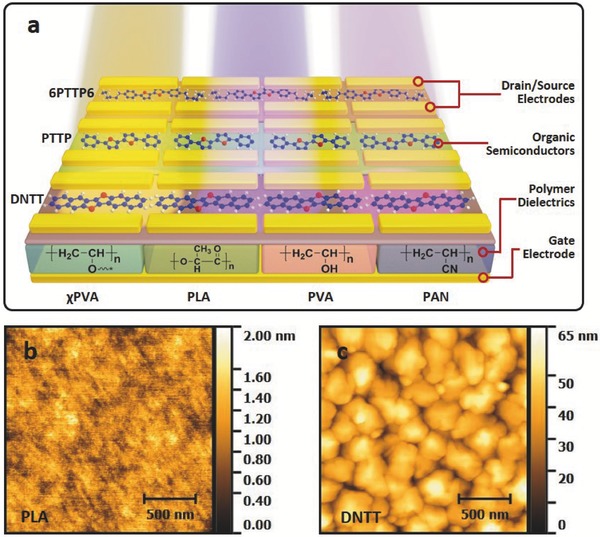
Schematic and AFM images of the OFETs. a) Construction scheme of the OFETs with a range of OSCs and polymer dielectrics, and AFM height image of b) PLA film and c) DNTT membrane deposited onto the PLA film.

The device geometry of the flexible top‐contact OFET is illustrated in **Figure** **1**a. Here, the OSCs include dinaphtho[2,‐b:2′, 3′‐f]‐thieno[3, 2‐b]thiophene (DNTT), 2,9‐didecyldinaphtho‐[2,3‐b:2,3‐f]thieno[3,2‐b]thiophene (C10‐DNTT), 5, 5′‐bis(4‐n‐phenyl)‐2, 2′‐bithiophene (PTTP), 5, 5′‐bis(4‐n‐ethylphenyl)‐2, 2′‐bithiophene(2PTTP2) and 5, 5′‐bis(4‐n‐hexylphenyl)‐2, 2′‐bithiophene (6PTTP6), *N*,*N*′‐bis (3‐(perfluoroctyl)propyl)‐1,4,5,8‐naphthalenetetracarboxylic acid diimide (8_3‐NTCDI). DNTT and C10‐DNTT were chosen due to its relatively high mobility, the PTTP series OSCs were adopted because they contain the same π‐conjugated core structure with different side chain lengths, and 8_3‐NTCDI was used because it is an n‐type OSC.^24^, ^25^ The dielectric polymers used in this work include poly(vinyl alcohol) (PVA), crosslinked PVA (χPVA), polylactide (PLA) and polyacrylonitrile (PAN). These polymers were selected because they are extensively used as dielectrics for flexible OFETs, and their functional groups are diversified.^14^, ^26^ The dielectric layers were fabricated with spin coating or dip coating for the different polymers. The thicknesses of the polymer films were measured by employing Surfcorder, and the fabricating parameters were further adjusted to form polymer films with thickness all around 1 µm. The roughness of these polymer dielectric membranes is on the order of nanometers (Figure 1b; Figure S1a,c,e, Supporting Information). Such smooth surfaces were essential to deposit on top high quality OSC films using thermal evaporation (Figure 1c; Figure S1b,d,f, Supporting Information).

**Figure 2:**
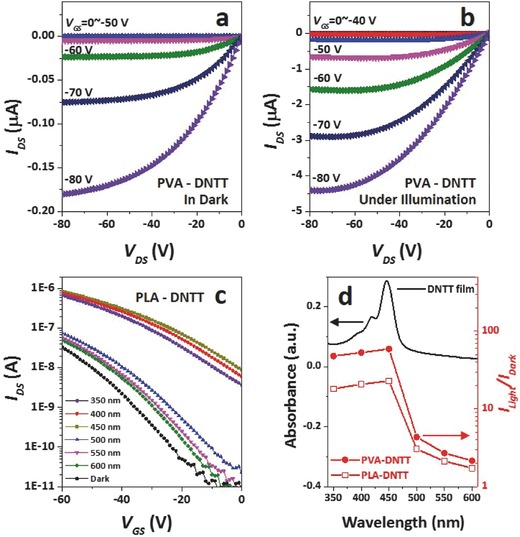
Response of the OFET performances to illumination. *I*_DS_ *–V*_DS_ characteristics of the PVA‐DNTT‐OFET a) in the dark and b) under illumination (white light, *P* = 50 mW cm^−2^), c) transfer characteristics of PLA‐DNTT‐OFET in the dark and under illumination with different wavelengths (*V*_DS_ = −60 V, *P* = 1 mW cm^−2^) and d) UV absorbance of DNTT membrane and *I*_light_ */I*_dark_ variations of PVA‐DNTT‐OFET and PLA‐DNTT‐OFET along with light wavelength (*V*_DS_ = −60 V, *V*_GS_ = −60 V, *P* = 1 mW cm^−2^).

The OFET characteristic curves, including source–drain curves (*I*_DS_ *–V*_DS_) and transfer plots (*I*_DS_ *–V*_GS_), were measured in the dark and under illumination with different power intensities or wavelengths. Generally, the OFETs exhibited a performance response to illumination. **Figure** **2**a,b shows the *I*_DS_ *–V*_DS_ curves of the OFET fabricated with a PVA dielectric and a DNTT semiconductor (PVA‐DNTT‐OFET) in the dark and under illumination, and the *I*_DS_ of the device obviously increased while illuminated by white light with an intensity of 50 mW cm^−2^. The photosensitive OFET performance is mainly attributed to the stimulation of illumination to the interfacial effect rather than the intrinsic photosensitivity of OSC, because as compared with PVA‐DNTT‐OFET, the DNTT‐OFET with the same OSC but a silica dielectric exhibited limited photosensitivity (Figure S2, Supporting Information). The same behavior was also observed for n‐type semiconductors. For example, photosensitivity of the PLA‐8_3‐NTCDI‐OFET is obviously stronger than that of 8_3‐NTCDI‐OFET with silica dielectric (Figure S3, Supporting Information). Furthermore, different electric performances were observed when the OFETs were exposed to illuminations with the same power intensity but with different wavelengths. Figure 2c presents the *I*_DS_ *–V*_GS_ curves of PLA‐DNTT‐OFET exposed to 1 mW cm^−2^ illuminations with wavelengths ranging from 350 to 600 nm. As compared to the dark measurement, the device showed a slight *I*_DS_ increase under illumination with wavelengths longer than 450 nm, and exhibited obvious *I*_DS_ increase when the light wavelength was shorter than 450 nm. Variations of the light‐to‐dark current ratio (*I*_light_/*I*_dark_) along with wavelength show “cut‐off” changes after 450 nm for both PLA‐DNTT‐OFET and PVA‐DNTT‐OFET (Figure 2d). Interestingly, the UV–Vis absorbance of the DNTT film also shows a peak at around 450 nm. When the illumination wavelength is below the peak, the energy of the incident photons into the DNTT exceeds the band gap energy of the semiconductor, leading to the generation of photoexcitons. These results indicate that the stimulation of illumination to the interfacial effect is achieved through the photogenerated hole–electron pairs in the OSCs.^21^ In the following research, white light with broad wavelengths, which covers the UV–Vis absorbance peaks of all used OSCs, is employed. Various OFETs with a series of polymer dielectrics and OSCs were investigated to reveal the general concept on how the molecular characteristic of both the polymer dielectrics and the OSCs influence the interfacial effect.

### 2.2 Effect of Polymer Dielectric Functional Group

**Figure 3:**
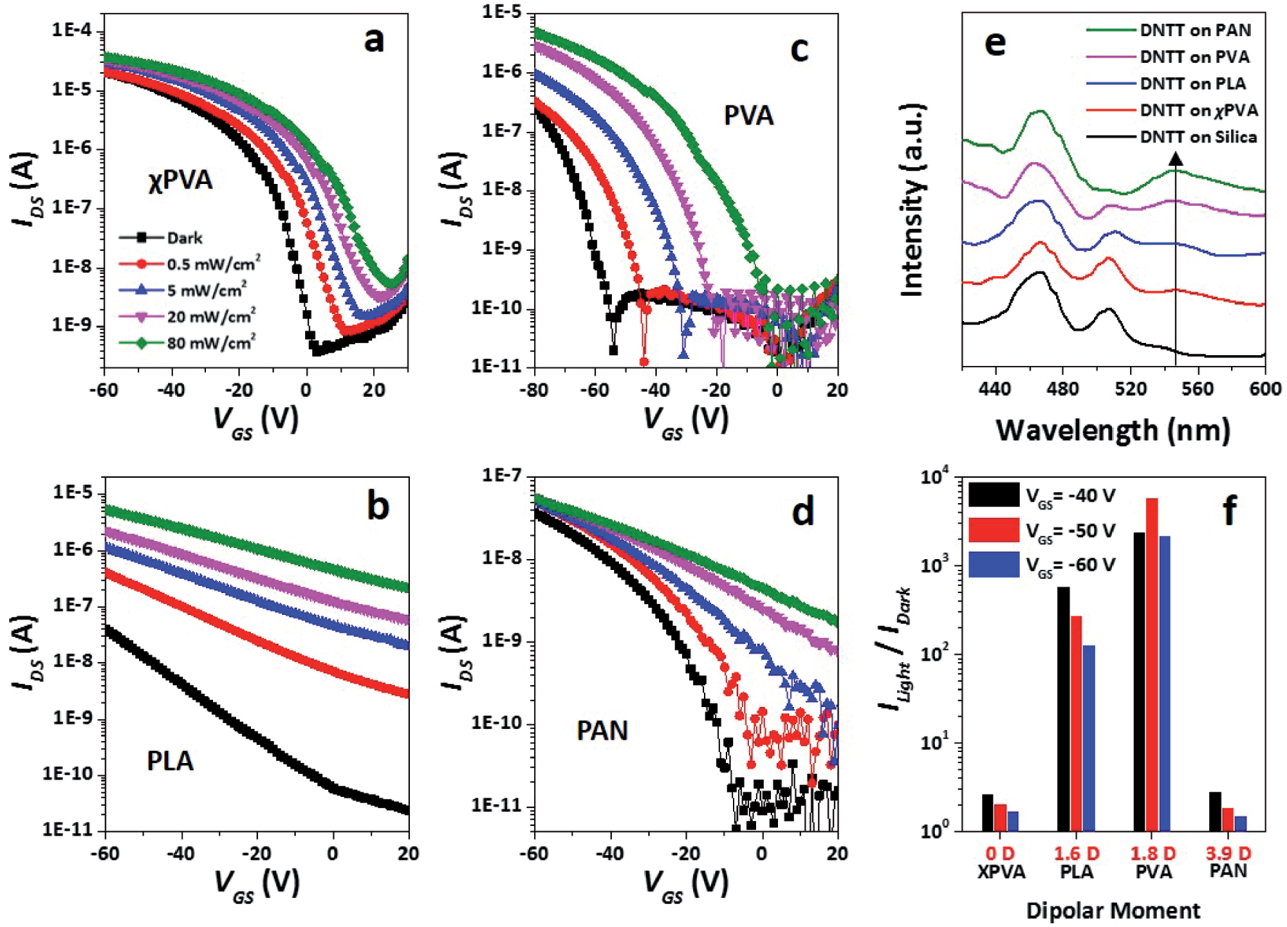
Effect of polymer dielectric functional group on OFET photosensitivity. Photosensitive transfer characteristics of DNTT‐OFETs with a) χPVA, b) PLA, c) PVA, and d) PAN dielectric (*V*_DS_ = −60 V), e) PL spectra of DNTT on different dielectric films (excitation at 340 nm), and f) *I*_light_ */I*_dark_ variations of different DNTT‐OFETs along with dipole moment of the polymer dielectrics (*V*_DS_ = −60 V, *P* = 80 mW cm^−2^).

OFETs with the same OSC but different polymer dielectrics showed significantly different photosensitivities. **Figure** **3** presents the photosensitive transfer curves of DNTT‐OFETs with four different polymers dielectrics. First, the *I*_DS_ of the OFETs tended to decrease from χPVA, PLA, PVA to PAN (Figure 3a–d). PLA, PVA, and PAN contain carbonyl, hydroxyl, and nitrile groups on the side, and the dipole moments of the functional groups are 1.6, 1.8, and 3.9 D, respectively. χPVA contains nonpolar ether groups in between the network structure instead of on the side. The dipole moment of χPVA functional groups is thus very weak, and approximatively denoted as 0 D. It should be noted that there might be few hydroxyl groups left in the χPVA film, and the actual dipole moment of χPVA should be slightly larger than 0 D. Photoluminescence (PL) spectra of DNTT membranes deposited on the different polymer dielectrics were recorded (Figure 3e). All the DNTT films show a pronounced 0–0 emission peak around 470 nm and a weak 0–1 peak around 510 nm.^27^ Interestingly, the broad band at 520–580 nm, which can be assigned to trapped excitons, exhibit an obvious intensity increase along with the dipole moment of the dielectric functional group. The results indicate that stronger polarity of functional group leads to stronger interfacial trapping to charge carriers in OSC.

Stronger interfacial trapping results in lower *I*_DS_, however, *I*_light_/*I*_dark_ of the OFETs shows different variation along with dipole moment as compared to the *I*_DS_.^13^ *I*_light_/*I*_dark_ for χPVA‐DNTT‐OFET (0 D) and PAN‐DNTT‐OFET (3.9 D) were found to be less than 10, while these for PLA‐DNTT‐OFET (1.6 D) and PLA‐DNTT‐OFET (1.8 D) were as high as 10^3^ to 10^4^ (Figure 3f). The Λ‐shaped variation can be explained as follows: for the OFETs with PLA or PVA dielectric, appropriate interfacial traps are formed due to their decent dipole moments, and distributions of charge carriers in the devices achieve dynamic equilibriums between transporting in the conducting channels and being trapped near the interfaces.^13^, ^22^ Photogenerated charge carriers then fill the traps and supplement the conducting channels, leading to significant enhancement of OFET performance under illumination compared to performance in the dark; for χPVA‐DNTT‐OFET with a low dipole moment, the interfacial trapping is rather weak and the conducting channel is fully filled with charge carriers.^11^, ^12^, ^21^ The *I*_DS_ of the device is high even in dark, so the effect of photoexcitons on the device performance is rather limited; while for PAN‐DNTT‐OFET which possesses a large dipole moment, photogenerated charge carriers would be continuingly confined by the strong interfacial traps, leading to remaining low *I*_DS_ under illumination. This finding reveals how the polymer dielectric functional group influences the interfacial effect.

### 2.3 Effect of OSC Side Chain

**Figure 4:**
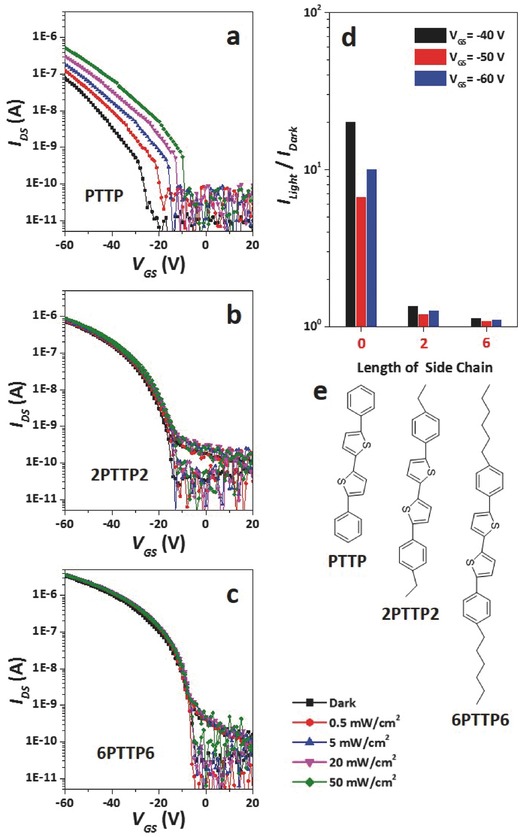
Effect of OSC side chain on OFET photosensitivity. Photosensitive transfer characteristics of a) PLA‐PTTP‐OFET, b) PLA‐2PTTP2‐OFET, and c) PLA‐6PTTP6‐OFET (*V*_DS_ = −60 V), d) *I*_light_ */I*_dark_ variations of different OFETs along with the length of the OSCs side chain (*P* = 50 mW cm^−2^), and e) molecular structure of the three OSCs. The light intensities of each colored curve are shown in the bottom right.

Side chain of OSC plays key roles in OFET fabrication and performance, such as increasing the OSCs solubility for solution‐processable fabrication, enhancing OSC molecular ordered alignment, or improving OFETs air stability, etc.^28^, ^29^, ^30^, ^31^ Investigation into how the OSC side chain influences the interfacial effect would also be interesting. OSCs of PTTP derivatives with different side chain lengths, including PTTP, 2PTTP2, and 6PTTP6, were then thermally evaporated onto PLA dielectrics to fabricate a series of OFETs. In the dark, the *I*_DS_ of PLA‐PTTP‐OFET is around 0.08 µA when *V*_DS_ and *V*_GS_ are both at −60 V, while the *I*_DS_ of PLA‐2PTTP2‐OFET and PLA‐6PTTP6‐OFET are 0.74 and 3.38 µA, respectively (**Figure** **4** a–c). As shown in Figure 4e, PTTP contains no side chain, while 2PTTP2 and 6PTTP6 contain alkane side chains with two and six carbon atoms, respectively. Longer side chain leads to higher *I*_DS_ for the OFETs. Furthermore, the transfer plots of PLA‐PTTP‐OFET in the dark and under illumination show an obvious difference, while the transfer plots of PLA‐2PTTP2‐OFET and PLA‐6PTTP6‐OFET present very limited changes. Figure 4d exhibits the variation of *I*_light_/*I*_dark_ along with the length of the OSC side chain, and the OSC without a side chain provides the OFET with the highest *I*_light_/*I*_dark_. It should be noted that the PTTP series OSCs exhibit very limited intrinsic photosensitivities themselves, and in comparison with Figure 4, the results indicate the OSC side chain possesses conspicuous impact on the interfacial effect. 6PTTP6 series molecules were observed to grown film with a vertical molecular mode by thermal evaporation, according to the results of X‐ray diffraction (XRD) and atomic force microscopy (AFM) measurements in previous literatures.^16^ The 6PTTP6 series films fabricated in this work exhibited the same XRD curves as compared with previous reports (Figure S4, Supporting Information). The nano morphologies of the 6PTTP6 series films also appeared similar with each other(Figure S5, Supporting Information). These results confirm the vertical orientation of the 6PTTP6 series molecules in the thermal evaporated fims. Based on these results, we suggest that the interfacial distance is the main mechanism for the effect of OSC side chain. In an OSC film, the conducting channel mainly consist of stacked π–π conjugated radicles, while the side chains are situated around the channel and generally located in between the channel and polymer dielectric.^29^, ^30^, ^31^ The length of the OSC side chain thus determines the distance between the OSC conducting channel and the interface. Apparently, an increased distance between the interface and the conducting channel leads to a weaker interfacial effect, and thus a longer side chain provides the OFET with a better *I*_DS_. Moreover, the interfacial trapping of PLA polar groups to the charge carriers is appropriately strong since PTTP possesses no side chain. Photogenerated charge carriers are then allowed to fill the traps and supplement the conducting channels, so that the photosensitivity of the PLA‐PTTP‐OFET is rather high. After side chains were introduced into the interface, the interfacial effect is then sharply weakened, leading to reduction of OFET photosensitivity. Therefore, longer OCS side chains lead to lower photosensitivity for the OFET.

The effect of OSC side chains on the interfacial effect was further confirmed by investigating DNTT series OFETs. C10‐DNTT possesses the same conjugated core with DNTT and decyl alkyl side chains, and the OSC was also observed to form evaporated films with a vertical orientation to substrates surfaces (Figure S6, Supporting Information).^31^ C10‐DNTT‐OFETs with PLA and silica dielectrics were then fabricated, and the OFETs both showed low photosensitivities. Comparing with the strong photosensitivity of PLA‐DNTT‐OFET, the results indicate that the longer side chains of C10‐DNTT also lead to lower photosensitivity for the PLA‐C10‐DNTT‐OFET. These findings are enough to make general conclusions on the effect of OSC side chains.

### 2.4 Linear Variation of the OFET Characteristics

**Figure 5:**
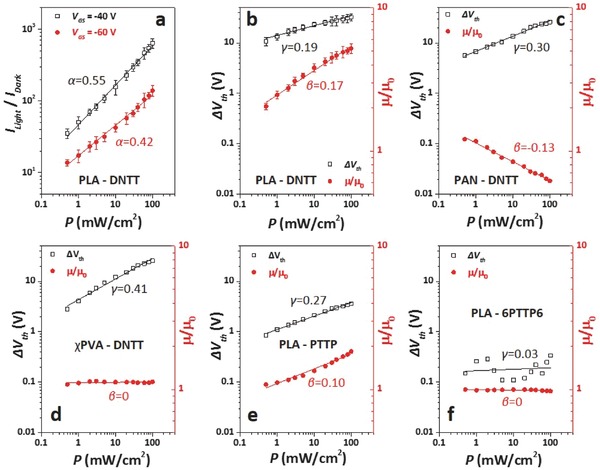
Linear fit of *I*_light_ */I*_dark_, μ, and *V*_th_ variations along with light intensity for the OFETs. a) Variations of *I*_light_ */I*_dark_ of PLA‐DNTT‐OFET, variations of μ and *V*_th_ of b) PLA‐DNTT‐OFET, c) PAN‐DNTT‐OFET, d) χPVA‐DNTT‐OFET, e) PLA‐PTTP‐OFET, and f) PLA‐6PTTP6‐OFET. Error bars in a) and b) represent standard errors from five OFETs.

Further analysis of the OFETs characteristics, including *I*_DS_, μ, and *V*_th_, as a function of light intensity (*P*) revealed interesting results. For a photoresponsive device, the light‐intensity‐dependent photosensitivity can be expressed as

(1) $$I_{\mathrm{Light}}/I_{\mathrm{Dark}}\propto P^{\alpha }$$

where the exponent, α, is a result of the complex processes of exciton generation, trapping, recombination, and so on within the devices.^32^, ^33^ A higher exponent value indicates better conversion efficiency of photons to device current. However, photoresponsive OFETs often exhibit *V*_GS_‐dependent photosensitivities. **Figure** **5**a presents logarithmic *I*_light_/*I*_dark_ changes along with logarithmic *P* for PLA‐DNTT‐OFET, and the curves both appear to be linear variations when *V*_GS_ is −40 and −60 V, respectively (*V*_DS_ = −60 V). The values of α are 0.55 and 0.42 for *V*_GS_ = −40 and −60 V. Higher α at *V*_GS_ = −40 V may be because the OFET operates at a more saturated state as compared to −60 V, so that photons can be more efficiently converted to device current. The value difference at different *V*_GS_ reminded us that α is not the most suitable exponent for revealing OFET photosensitivity.

As comparing with *I*_DS_, μ, and *V*_th_ are OFET characteristics that are less dependent on operating *V*_GS_.^34^, ^35^ The μ and *V*_th_ changes as a function of *P* were then analyzed. Figure 5b presents logarithmic variations of *μ/μ*_0_ (μ_0_ is μ of OFET in the dark) and ∆*V*_th_ (*V*_th_ minus *V*_th_ of OFET in the dark) along with logarithmic *P* for PLA‐DNTT‐OFET. Interestingly, the both variations also exhibited linear curves. We assume

(2) $$\mu /\mu_{0}\propto P^{\beta }$$

(3) $$\Delta V_{\mathrm{th}}\propto P^{\gamma }$$

where β and γ are nonunity exponents defined here to reveal rather instinct OFET photosensitivity. As we know for an OFET, the relationship between saturated *I*_DS_ (*I*_DS_ *_‐_*_sat_), μ, and *V*_th_ can be expressed as

(4) $$I_{\mathrm{DS‐sat}}=\frac{W}{2L}C_{i}\mu {\left(V_{\mathrm{GS}}-V_{\mathrm{th}}\right)}^{2}$$

where *W* and *L* are the width and length of the OFET channel, respectively, and *C*_i_ is gate‐channel capacitance per unit area.^36^ For a given OFET, *WC*_i_ */2L* is a constant, and combining Equations (1), (2), (3), (4) gives

(5) α=β+2γ

Figure 5a shows that α = 0.55 when the OFET operated in saturated model, while β = 0.17 and γ = 0.19 are obtained from Figure 5b. These values are perfectly consistent with Equation (5), indicating that the photoinduced *I*_DS_ change in the PLA‐DNTT‐OFET is a result of both μ and *V*_th_ variations. Considering the μ and *V*_th_ changes are the results of the effect of shallow and deep traps as previously reported, we realized that the both traps were present in PLA‐DNTT‐OFET, and are stimulated by illumination.^22^

The changes of μ and *V*_th_ are not always simultaneous for the OFETs, e.g., the PAN‐DNTT‐OFET exhibited a photoinduced ∆*V*_th_ increase and *μ/μ*_0_ decrease (Figure 5c), while χPVA‐DNTT‐OFET showed a photoinduced ∆*V*_th_ increase, but almost no *μ/μ*_0_ change (Figure 5d). First, the photoinduced μ decrease in Figure 5c could be explained as the result of a photoinduced *V*_th_ positive shift.^20^ Taking a p‐type OFET as an example, electrons from the photogenerated electron–hole pairs transfer across the semiconductor/dielectric interface with the assistance of the positive gate field at the beginning of the transfer curves measurements (the transfer curves of the OFETs were measured from positive to negative *V*_GS_ region), leaving extra holes in the OFET conducting channel, which makes the OFET easier to turn on, namely the *V*_th_ positive shift. Meanwhile, the transferred electrons in the dielectric act as extra shallow traps to further slowdown the hole transportation in the semiconducting channel. The density of the interfacial shallow traps increases, leading to a decrease μ. The relationship between μ and shallow trap density can be expressed as

(6) $$\mu \approx \mu_{0}\frac{1}{1+\Delta N/N_{0}}$$

where *N_0_* is the initial density of the shallow traps and ∆*N* is the increased density, which can be expressed as

(7) $$\Delta N\approx C_{i}\Delta V_{\mathrm{th}}/e$$

where *e* is the elementary charge.^20^ Combining Equations (6) and (7) gives

(8) $$\mu /\mu_{0}\approx \frac{1}{1+\frac{C_{i}}{eN_{0}}\Delta V_{\mathrm{th}}}={\left(k\Delta V_{\mathrm{th}}+1\right)}^{-1}$$

where *k* = *C*_i_/*eN*_0_  is a constant for a given OFET. Equation (8) reveals that a photoinduced ∆*V*_th_ increase could result in a μ decrease. On the other hand, the photogenerated holes in semiconducting channel can also fill the interfacial shallow traps, and simultaneously increases μ for the device. The two opposite tendencies thus synchronously affect the OFETs and provide the devices with diversified μ variations. Second, the OFETs in Figure 5b–d possess the same OSC but different functional groups of polymer dielectrics. The devices show different μ changes but consistent *V*_th_ positive shift, indicating that the influence of polymer functional group on the interfacial effect is realized by changing the state of the shallow traps rather than the state of the deep traps.

Figure 5e,f presents the photo‐dependent μ and *V*_th_ variations of the PLA‐PTTP‐OFET and the PLA‐6PTTP6‐OFET. The former presents photoinduced μ and *V*_th_ positive shift, while the later exhibits very limited μ and *V*_th_ variations along with light intensity. These results indicate that the effect of the OSC side chain on the shallow trap and the deep trap are both obvious. A longer side chain leads to weaker shallow and deep traps. It also should be noted that for our p‐type OFETs, *V*_th_ positive shifted along with light intensity, which means that the hole density increased under illumination. This finding suggests that the lowest unoccupied molecular orbital energy levels of the OSCs march with these of the polymer dielectrics, while the highest occupied molecular orbital energy levels of the OSCs are far higher than those of the dielectrics.^21^, ^25^, ^28^ Electrons in photoexcitons are thus allowed to transfer from the OSCs to the polymer dielectrics, leaving holes conducting in the OSCs, which make the OFETs exhibit a photoinduced *V*_th_ positive shift.

### 2.5 Mechanism of the Interfacial Effect and Photosensing Performance Optimization

**Figure 6:**
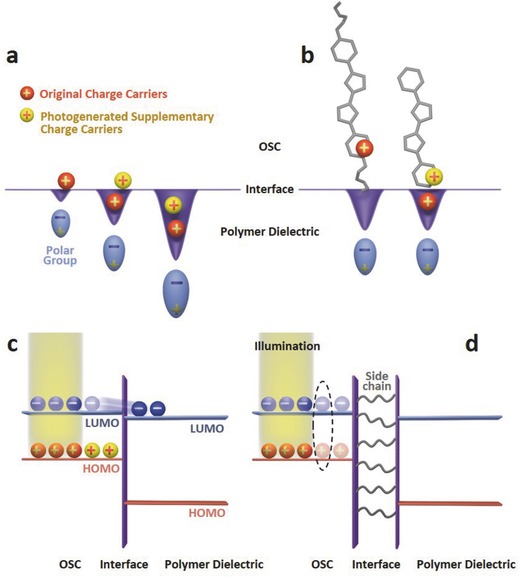
Schematic illustration of the interfacial effect between the OSC and the polymer dielectric in OFETs. a) Polar groups of polymer dielectric induce shallow traps with different energy levels on the interface, and only the traps with appropriate energy levels (in the middle) result in an obvious photosensitive μ change for the OFET; b) the OSC side chain hinders the shallow trap and leads to debilitated photoinduced μ variations for the OFET; c) for p‐type OFETs, electrons in photogenerated hole–electron pairs transfer from the OSC to the polymer dielectric layer and be deeply trapped, leaving extra holes in the OSC conducting channel, and the corresponding OFET exhibits *V*_th_ positive shift under illumination; d) OSC side chain hinders electron transfer, resulting in debilitated photoinduced *V*_th_ variations for the OFET.

Detailed knowledge about the interfacial effect is revealed based on the systematic investigation. First, the functional groups of polymer dielectrics could shallowly trap the charge carriers in the conducting channels of the OSCs, thus reducing the charge carrier transportation rate. The trapping degree is significantly affected by the dipole moment of the polymer dielectric functional groups and the distance between the functional groups and the OCS conducting channels. Thus, stronger dipole moment and shorter side chain leads to stronger shallow traps and a lower μ for the OFET (**Figure** **6**a,b). Under illumination, the ability of the shallow traps to capture charge carriers would be reduced and saturated after a large amount of photoexcitons are generated. The transportation rate of extra photogenerated charge carriers would not be affected by those shallow traps, and thus for the PLA‐PTTP‐OFET and the PLA‐DNTT‐OFET (Figure 5b,e), μ increases along with light intensity. Long OSC side chains and low polarity of polymer functional group leads to weak shallow traps on the devices interfaces, and thus the χPVA‐DNTT‐OFET and the PLA‐6PTTP6‐OFET exhibit limited μ variations (Figure 5d,f). Additionally, polymer dielectrics with strong polar groups, for example PAN, could form strong shallow traps. The effect of the strong shallow trap cannot be fully saturated by the photoexcitons, and thus the PAN‐DNTT‐OFET maintains a relatively low μ even under illumination.

Second, electrons from photogenerated hole–electron pairs could transfer across the OSC/dielectric interface with the assistance of the gate field, and become deeply trapped in the polymer dielectrics. Extra holes are left in the OSC conducting channel, leading to a *V*_th_ positive shift for the p‐type OFET (Figure 6c). Long OSC side chain could hinder the electron transfer process, and the hole–electron pairs recombine in the OSC (Figure 6d), which is why *V*_th_ of PLA‐6PTTP6‐OFET exhibits limited change along with light intensity (Figure 5f). Moreover, deeply trapped electrons in polymer dielectrics induce extra shallow traps, which could result in a μ decrease for the OFET (Figure 5c).^20^ It should be noted that this explanation is mainly applicable to OSCs with limited intrinsic photosensitivity and side chain efficient separates the semiconducting channel and dielectric. Otherwise, OFETs based on OSCs with rather long side chains could still exhibit obvious photosensitivities.^37^

Several other factors that may result in photoresponsive behavior for the OFETs were excluded in this work, including ambient atmosphere and capacitance change in the dielectric. The complicated interactions between oxygen and water in ambient atmosphere with OSCs were often considered as a major effect on OFET performance.^38^ Photosensitivities of the PLA‐DNTT‐OFET were thus measured both in air and in vacuum (Figure S7, Supporting Information). A stronger photoresponsivity of the device was observed in vacuum compared to that in air, indicating that the photosensitivities of our devices is not induced by oxygen or water in ambient atmosphere. Capacitance variation of the PLA film, along with light intensity, was also found to present a limited change (Figure S8, Supporting Information). The above results indicate that the diversified photosensitivities of the OFETs are determined by the states of interfacial effects in the devices.

**Table** **1** shows the effect of the polymer dielectric polar group and the OSC side chain on the interfacial effect, and how the state of the interfacial effect influences the OFET photosensing performance. Table S1 in the Supporting Information exhibits some basic parameters of the OFETs investigated in this work. OFETs with controllable electric performance can be fabricated by selecting appropriate OSCs and polymer dielectrics, e.g., Figure 3 presents OFETs with *I*_DS_ raging from dozens of nanoamperes to scores of microamperes. Furthermore, PLA‐6PTTP6‐OFET exhibits almost no photosensitivity due to the long side chain of 6PTTP6, and *I*_light_/*I*_dark_ of the PAN‐DNTT‐OFET is less than 10 due to the strong polarity of the PAN functional groups. PLA‐DNTT‐OFET and PVA DNTT‐OFET, on the other hand, show *I*_light_/*I*_dark_ up to 10^4^ thanks to the lack of the OSC side chains and appropriate polarity of both the PLA and PVA functional groups. Therefore, photosensitivity optimization of OFETs can be achieved by modulating the polar groups of the polymer dielectrics and the OSC side chains.

**Table 1:** The effect of the polymer dielectric polar group and the OSC side chain on the interfacial effect, and optimization of the OFET photosensing performance by utilizing the mechanism

Parameter	Interfacial effect		Photosensing performance	
	Shallow trap	Deep trap	*μ/μ*_0_	∆*V*_th_
Polarity of polymer dielectric functional group	Stronger polarity leads to stronger shallow trap	Limited influence	Moderate polarity leads to the highest *μ/μ*_0_	Limited influence
Length of OSC side chain	Shorter side chain leads to stronger shallow trap	Shorter side chain leads to stronger deep trap	Shorter side chain leads to higher *μ/μ*_0_	Shorter side chain leads to higher ∆*V*_th_, and photoinduced *V*_th_ positive shift results in μ decrease

### 2.6 Photostimulated Synapse Emulating OFETs

**Figure 7:**
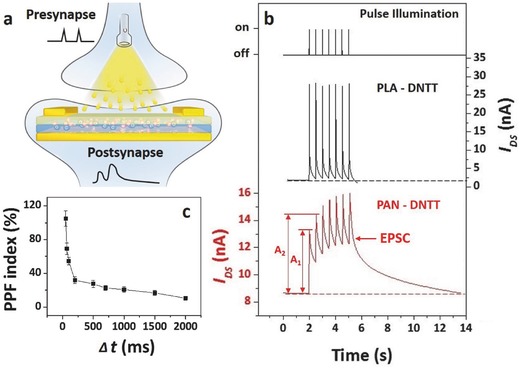
Photostimulated synaptic behaviors of the OFETs. a) Schematic of applying the OFETs to emulate photostimulated synaptic behaviors, b) the output *I*_DS_ signals of the PLA‐DNTT‐OFET (*V*_GS_ = −20 V, *V*_DS_ = −60 V) and the PAN‐DNTT‐OFET (*V*_GS_ = −40 V, *V*_DS_ = −60 V) exposed to input light synaptic trains with light on time of 10 ms (*P* = 1 mW cm^−2^) and off time of 500 ms, and c) PPF index, defined as 100% × (A2−A1)/A1, as a function of interspike interval. Error bars in c) represent standard errors from five measurements.

Furthermore, the OFETs with moderate strong interfacial effect exhibit EPSC‐like photoresponse behavior, and thus can be applied as photostimulated synapseemulator. Synapses are among the most important functional units of our brain which is an energy‐efficient and event‐driven information processing system, and can outperform supercomputers in many tasks.^39^, ^40^, ^41^ Photostimulated synapseemulator is also promising for brain‐inspired electronics and human–machine interface, etc.^8^ The important working principles of a synapse is the transformation of input synaptic trains into appropriate output signals, namely EPSC. Here, our OFETs realized mimicking synaptic signal transformation by outputting additive photocurrent caused by repeated light pulses (**Figure** **7**a). Figure 7b presents the output *I*_DS_ signals of the PLA‐DNTT‐OFET and the PAN‐DNTT‐OFET under input light pulse. While the PLA‐DNTT‐OFET shows a stable pulse *I*_DS_ output signal which corresponds to the input light pulse, the PAN‐DNTT‐OFET exhibits a typical EPSC output signal. The different behaviors of the two OFETs are assigned to the different degrees of the interfacial effect. The PAN‐DNTT‐OFET possesses a strong interfacial effect. The photogenerated charge carriers form a short time light stimulation cannot fulfill the interfacial traps in the OFET, and thus subsequent light stimulations can continually increase the device *I*_DS_. After the light stimulation, it also takes rather long time for the traps to release charge carriers and to reach the equilibrium device *I*_DS_ in the dark. The *I*_DS_ of the device is observed to increase when the second spike closely follows a previous spike, indicating the PAN‐DNTT‐OFET is able to emulate the paired‐pulse facilitation (PPF) behavior of synapses.^41^ Figure 7c exhibits that the PPF index (100% × (A2 − A1)/A1, where A1 and A2 are the heights of the first and second EPSC peaks in Figure 7b) decreases with increase of time interval (∆*t*). The PPF index decreases from 105% to 10% when the time interval increases from 50 to 2000 ms, indicating our synapse‐emulating OFET possesses a good short‐term potentiation.

## 3 Conclusion

We systematically investigated the “hidden” interfacial effect between a series of polymer dielectrics and OSCs in flexible OFETs by subtly utilizing illumination to stimulate the effect, and to characterize the response of the effect by measuring the photoinduced OFETs characteristics changes. Based on the investigation, the influence of molecular characteristic of the OSCs and the polymer dielectrics on the two interfacial effect models, both shallow trap and deep trap, were revealed in detail. We also demonstrated that the interfacial effect dominates the OFET photosensing performance. Specifically, stronger polarity of polymer dielectric functional groups and shorter OSC side chains lead to a stronger shallow trap, and a lower OFET μ value. Photogenerated charge carriers would fill the shallow traps and increase μ, giving photosensitivity to the devices. Shorter OSC side chains also lead to stronger deep traps. Electrons from photogenerated hole–electron pairs can transfer across the OSC/dielectric interface and become deeply trapped in polymer dielectrics, leaving holes in the OSCs conducting channels, which leads to photoinduced *V*_th_ positive shift. The photoinduced μ and *V*_th_ changes exhibited logarithmically linear variation along with light intensity, which allows us to express the processes in equations. Two exponents, β and γ, were defined to describe the intrinsic photosensing performance of the OFETs. Moreover, photosensors with controllable performances can be obtained by utilizing the mechanism, e.g., *I*_light_/*I*_dark_ of the OFETs can be tuned from below 10 to beyond 10^4^. OFETs with moderate strong interfacial effect can also exhibit EPSC‐like photoresponse behavior. Photostimulated synapse emulator is thus obtained and applicable for brain‐inspired electronics and human–machine interface, etc. Given a comprehensive understanding on organic electronic interface effect, our work provides predictions for OFET characteristics with given OSCs and polymer dielectrics, and opens compelling strategies for low‐cost easy‐fabrication of flexible photosensors.

## 4 Experimental Section

*Materials and Device Fabrication*: The OSCs, including DNTT, C10‐DNTT, PTTP, 2PTTP2, 6PTTP6, and 8_3‐NTCDI were synthesized as literature reported, followed by vacuum sublimation purification.^24^, ^25^, ^42^, ^43^ PLA (3052D) was purchased from Natureworks Co., Ltd., and further purified by dissolution in chloroform and precipitated with methanol. PVA with molecular weight around 16 000 was purchased from Alfa Aesar Co., Ltd., and the crosslinking agent for PVA, ammonium dichromate, was purchased from Aladdin Co., Ltd. PAN with molecular weight around 150 000, octadecyltrichlorosilane (OTS), and the OFET releasing agent (tridecafluoro‐1, 1, 2, 2‐tetrahydrooctyl) trichlorosilane (FOTS) were purchased from Sigma‐Aldrich Co., Ltd.

OFETs were made using silicon wafers as template substrate. FOTS chloroform solution (1% v/v) was used to modified the substrates by spin coating at 3000 rpm for 20 s, followed by sonication cleaning in chloroform for 30 min. With the FOTS release layer, the OFET could be peeled off from silicon substrates easily after the fabrication process was completed.^7^, ^11^ 100 nm gold gate electrodes were thermally evaporated onto the substrate after the deposition of the release layer. Different polymer dielectric films were then fabricated as following: (a) PLA dielectric films were fabricated by dip coating 50 g L^−1^ PLA chloroform solution at a speed of 20 µm s^−1^ (Dip Coater, Shanghai SANYAN Co., Ltd., SYDC‐100H); (b) PAN dielectric films were fabricated by dip coating 40 g L^−1^ PAN in *N*,*N*‐dimethylformamide solution at a speed of 20 µm s^−1^ at 60 °C; (c) PVA dielectric films were fabricated by spin coating 50 g L^−1^ PVA in aqueous solution at 3000 rpm for 1 min; and (d) ammonium dichromate (mass ratio of ammonium dichromate to PVA is 1:6) was added into 50 g L^−1^ PVA aqueous solution, and the mixture solution was spin coated to form film at 3000 rpm for 1 min. The film was then crosslinked under exposure of 254 nm UV light for 10 min. The resulted films were dried at 60 °C in air overnight. The thicknesses of the polymer dielectrics were measured with a Surfcorder (Kosaka Laboratory Co., Ltd.). After that, 50 nm of the OSCs, including DNTT, C10‐DNTT, PTTP, 2PTTP2, 6PTTP6, and 8_3‐NTCDI were deposited at the rate of 0.3 Å s^−1^ by vacuum thermal evaporation (*T*_substrate_ = 60 °C, *P* ≈ 5 × 10^−4^ Pa). 100 nm gold source–drain electrodes were thermally evaporated through a shadow mask to form top‐contact OFETs. The channel length (*L*) and width (*W*) are 0.2 and 6.0 mm, respectively. OFETs with silica dielectric were fabricated for experiment control. Silicon wafers with 300 nm of thermally grown silica layers were immersed in OTS toluene solution (1.2% v/v) at room temperature for 3 h, followed by washed with toluene and ethanol. Subsequently, the substrates were annealed at 120 °C for 20 min and cleaned by sonication in toluene for 30 min. The OTS‐treated substrates were washed with ethanol and water and dried in flowing pure nitrogen. The deposition processes of OSCs and gold electrodes for OFETs with silica dielectric were as the same as mentioned above.

*Device Characterizations*: The surface morphologies of all the polymer dielectric films and OSC thin films were observed by using AFM (SEIKO SPA‐300HV) operated in tapping mode. UV–vis spectrum of the DNTT film on quartz substrate was recorded on a Cary‐60 UV–vis spectrophotometer (Agilent Technologies Co., Ltd.). PL spectra of the DNTT membranes on various dielectric films were measured by using a Lumina Fluorescence Spectrometer (Thermo Fisher Co., Ltd.), and the DNTT membranes were fabricated with thickness of 30 nm. XRD measurements were carried out using DX‐2700 X‐ray diffractometer (Dandong Haoyunan Instrument Co. Ltd) with Cu Kα radiation (1.54 Å) at 40 kV and 50 mA.The capacitance measurement of PLA film was taken by an inductance‐capacitance‐resistance measuring instrument (LCR meter) (TH2827C, Changzhou Tonghui Electronics Co., Ltd.). Electrical performances of the OFETs were characterized with a Keithley 4200‐SCS Semiconductor Parameter Instrument, and the measurements were carried out under vacuum unless otherwise indicated. For the characterization of the OFETs under illumination with different intensities and wavelengths, a 300 W xenon lamp (wavelength 200–2000 nm) filtered by a double grating monochromator (Omno330150, Beijing NBeT Co., Ltd.) was employed. The illumination intensities were measured by an optical power meter (Thorlabs Co., Ltd. PM100D). The light was illuminated from the top side of the OFETs.

## Conflict of Interest

The authors declare no conflict of interest.

## Supporting information

Supplementary
