# Supplementary material for: Pursuing Polymer Dielectric Interfacial Effect in Organic Transistors for Photosensing Performance Optimization
Source: Adv Sci (Weinh). 2017 Oct 16;4(12):1700442. doi: 10.1002/advs.201700442 (PMC5737237; doi:10.1002/advs.201700442)
Supplement: Supplementary file 1 — Supplementary [file ADVS-4-na-s001.pdf]

Copyright WILEY-VCH Verlag GmbH & Co. KGaA, 69469 Weinheim, Germany, 2017.

Supporting Information

for *Adv. Sci.,* DOI: 10.1002/advs.201700442

Pursuing Polymer Dielectric Interfacial Effect in Organic Transistors for Photosensing Performance Optimization

*Xiaohan Wu, Yingli Chu, Rui Liu, Howard E. Katz, and Jia Huang**

Supporting Information for

Pursuing Polymer Dielectric Interfacial Effect in Organic Transistors for Photosensing Performance Optimization

*Xiaohan Wu, Yingli Chu, Rui Liu, Howard E. Katz and Jia Huang**

Figure s1. Atomic Force Microscope height image of a) χPVA film, b) DNTT film deposited on χPVA, c) PVA film, d) DNTT film deposited on PVA, e) PAN film and f) DNTT film deposited on PAN.

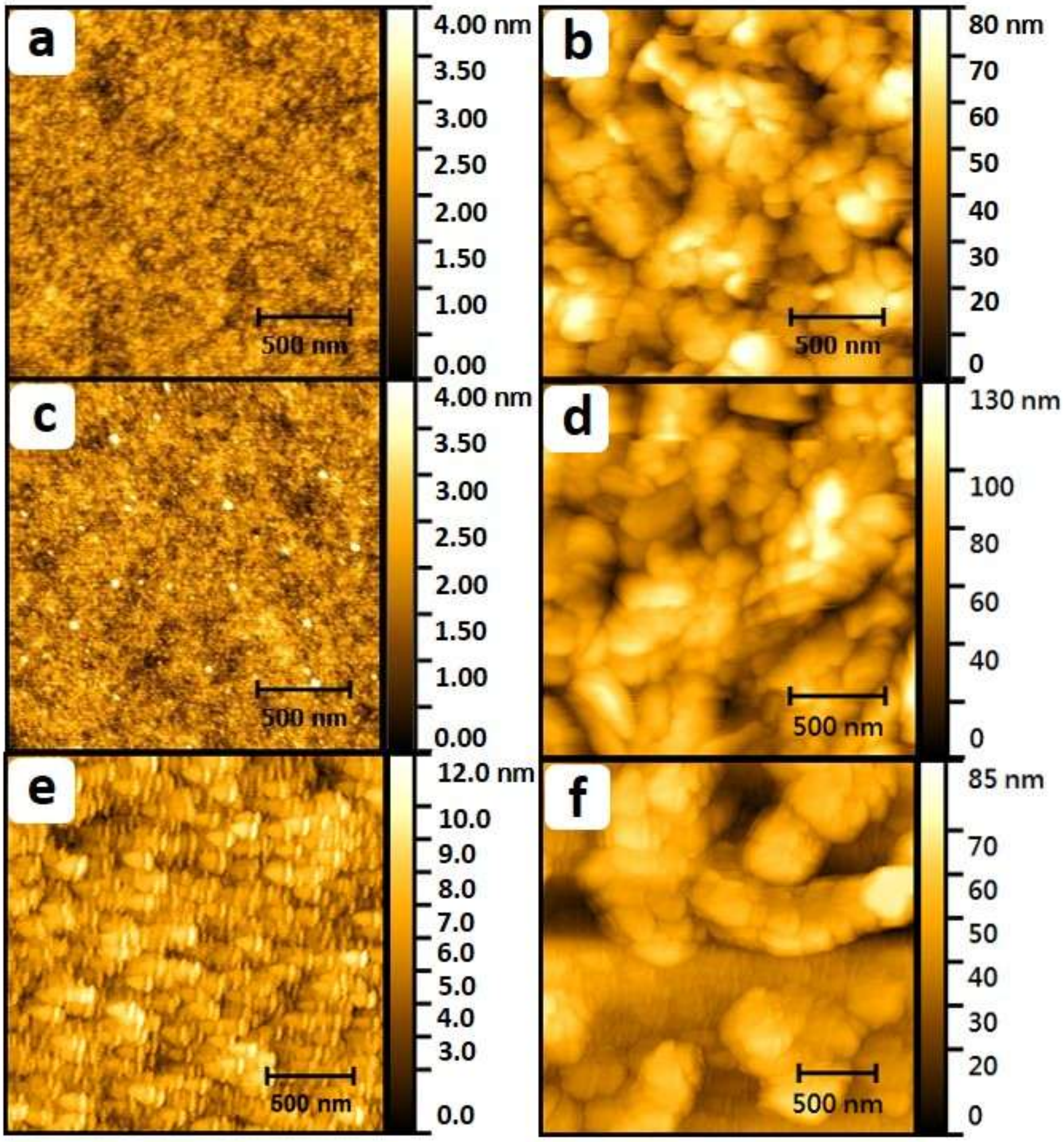

Figure s2. Photosensitivity of DNTT-OFET with a) silica and b) PVA dielectric.

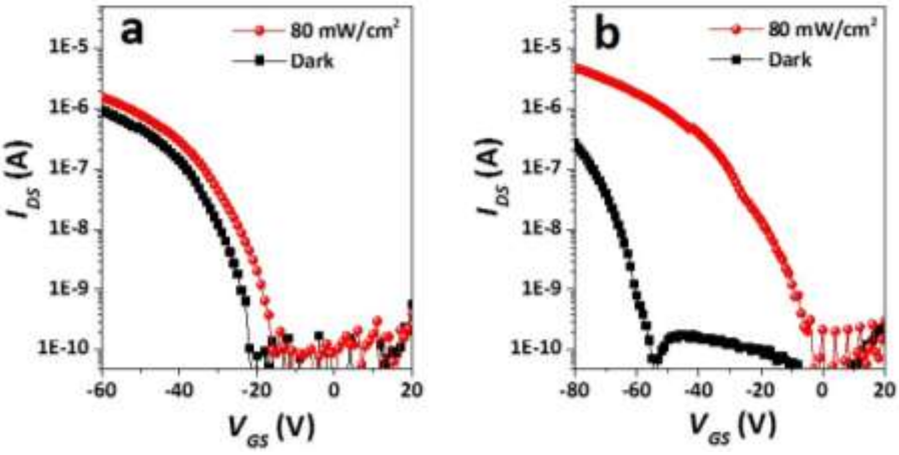

Figure s3. a) Molecular structure of 8_3-NTCDI, amd photosensitivity of 8_3-NTCDI-OFET with b) silica dielectric and c) PLA dielectric.

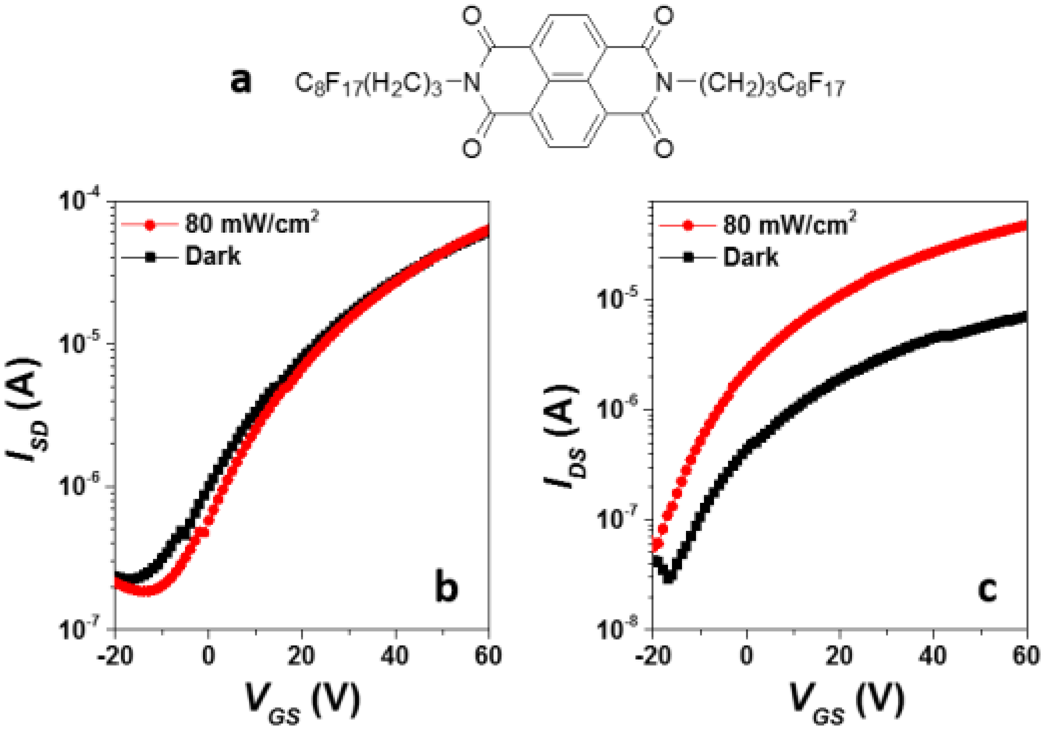

Figure s4. XRD cureves of PTTP, 2PTTP2 and 6PTTP6 films.

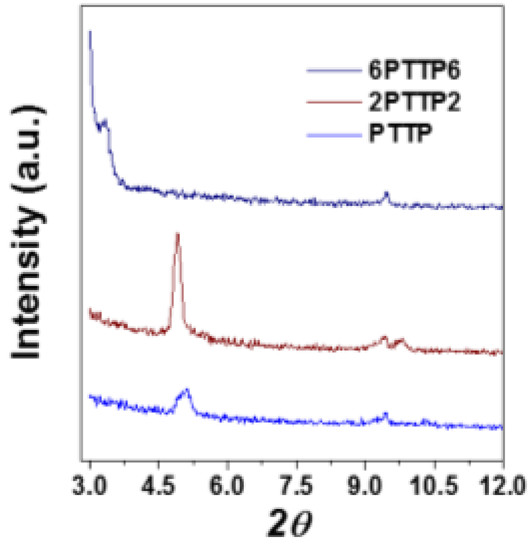

Figure s5. AFM images of PTTP, 2PTTP2 and 6PTTP6 films.

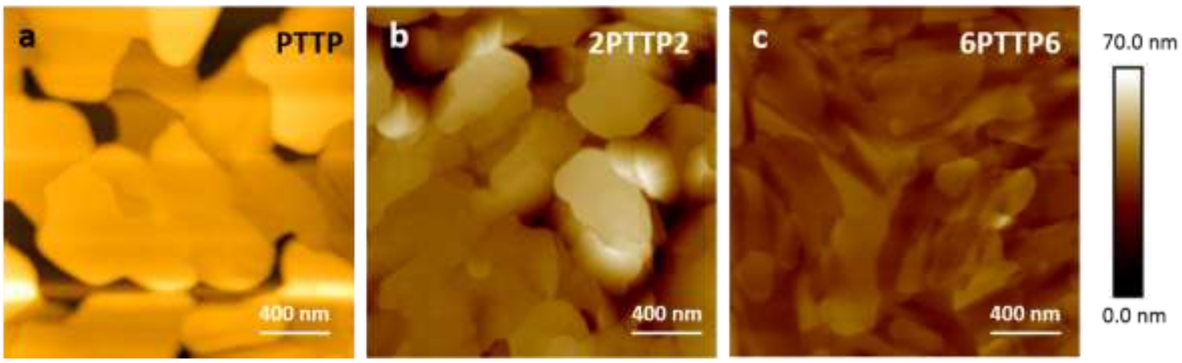

Figure s6. a) Molecular structure of DNTT and C10-DNTT, and photosensitivity of C10-DNTT-OFET with b) silica and c) PLA dielectric.

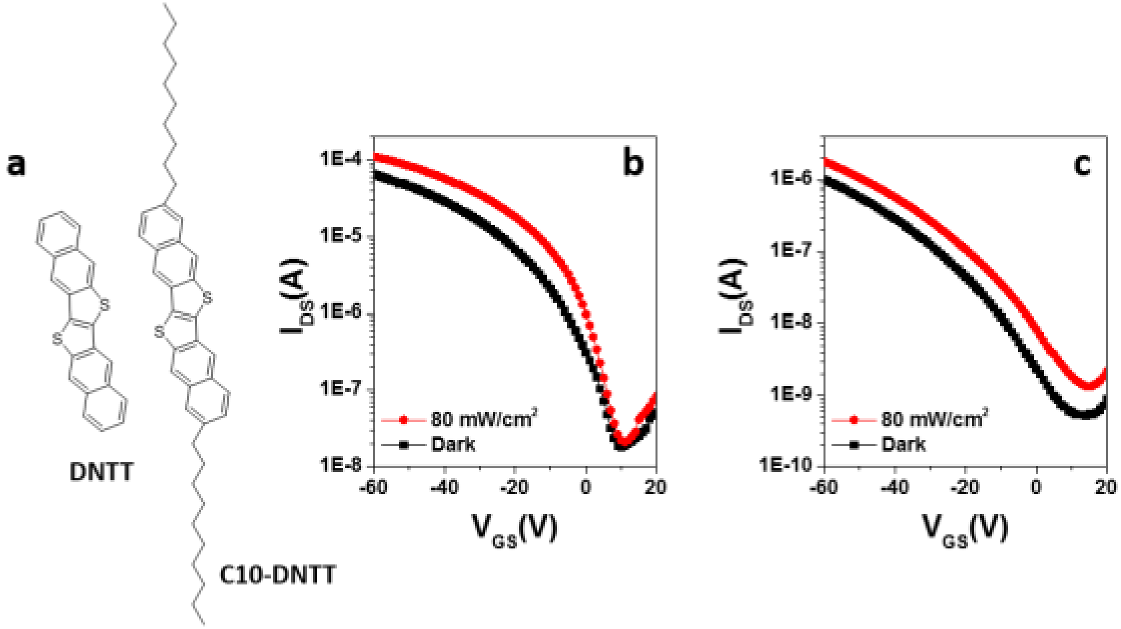

Figure s7. Photosensitivity behavior of PLA-DNTT-OFET a) in air and b) under vacuum.

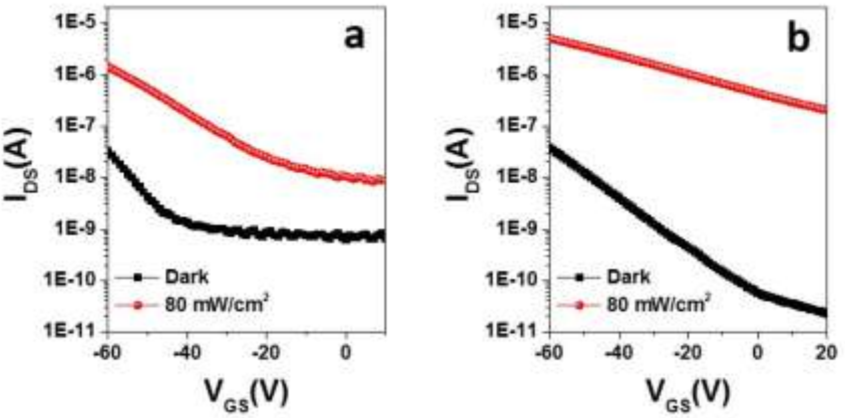

Figure s8. Capacitance variation of PLA film between gold top and bottom electrodes along with light intensity.

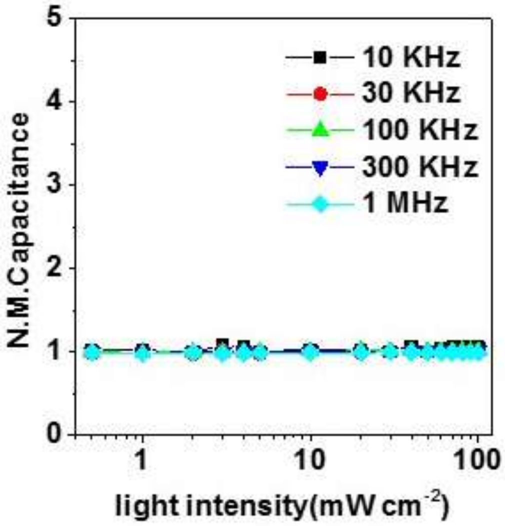

Table s1. Parameters of the OFETs investigates in this work. [a]light means under white light with intensity of 50 mW/cm^2 for the PTTP series OFETs, and 80 mW/cm^2 for the other OFETs. [b]V_{DS} = -60V, and V_{GS} = -60V.

OFET	μ dark (cm^{2}V^{-1}s^{-1})	μ light[a] (cm^{2}V^{-1}s^{-1})	V_{th} dark (V)	V_{th} light[a] (V)	I_{light} / I_{dark}[a, b]
χPVA-DNTT-OFET	0.354	0.356	-1	19	1.7
PLA-DNTT-OFET	0.0022	0.012	-37	-5	130.9
PVA-DNTT-OFET	0.055	0.087	-63	-22	2237.6
PAN-DNTT-OFET	0.0013	0.0009	-8	17	1.5
PLA-PTTP-OFET	0.009	0.017	-22	-16	10.1
PLA-2PTTP2-OFET	0.027	0.028	-18	-17	1.3
PLA-6PTTP6-OFET	0.082	0.083	-7	-6	1.1
